# Decision-Making Approaches Used to Limit Potentially Nonbeneficial Life-Prolonging Interventions

**DOI:** 10.1001/jamanetworkopen.2025.60260

**Published:** 2026-02-20

**Authors:** Jason N. Batten, Sofia Weiss Goitiandia, Julia K. Axelrod, Helen O. Chernicoff, Ariadne A. Nichol, Lorraine M. Pereira, Jacob A. Blythe, Jacqueline M. Kruser, Elizabeth W. Dzeng

**Affiliations:** 1 Department of Anesthesiology and Perioperative Medicine, University of California, Los Angeles, Los Angeles; 2 Division of Hospital Medicine, Department of Medicine, University of California, San Francisco, San Francisco; 3 Department of Palliative Medicine, Swedish Cherry Hill, Seattle, Washington; 4 School of Medicine, University of California, San Diego, San Diego; 5 Harvard Medical School, Boston, Massachusetts; 6 Department of Radiology, Massachusetts General Hospital, Boston; 7 Department of Medicine, Division of Allergy, Pulmonary, and Critical Care; University of Wisconsin-Madison, Madison; 8 Philip R. Lee Institute for Health Policy Studies, University of California, San Francisco, San Francisco; 9 Cicely Saunders Institute, King’s College London, London, England

## Abstract

**Importance:**

Professional society policy statements recommend that clinicians limit (ie, withhold or withdraw) potentially nonbeneficial life-prolonging interventions by (1) achieving a shared decision with patients or surrogates or (2) initiating an institutional process to address disagreement with patients or surrogates. However, in the context of a health care system with a default tendency toward life prolongation, it is unclear whether clinicians rely entirely on these recommended approaches or resort to alternate approaches.

**Objective:**

To characterize the range of decision-making approaches clinicians report using to limit potentially nonbeneficial life-prolonging interventions.

**Design, Setting, and Participants:**

This qualitative study was conducted at 3 tertiary academic medical centers in Washington and California. Clinicians were sampled from emergency department, medical ward, medical intensive care unit, geriatrics, and palliative care services between February 2018 and June 2022 for in-depth, semistructured interviews. Results were analyzed between August 2023 and May 2025.

**Main Outcomes and Measures:**

After qualitatively analyzing interviews to identify decision-making approaches, we developed a framework of approaches that categorized each as a recommended or alternate approach.

**Results:**

We conducted 101 interviews (53 attending physicians [52%], 16 trainee physicians [16%], 6 advanced practice clinicians [6%], 21 nurses [21%], 3 chaplains [3%], and 2 social workers [2%]; 59 women [58%], 42 men [42%]; mean age, 42 years [range, 27-74 years]; mean years of experience, 14 [range, 1-52]). We identified 6 decision-making approaches: (1) providing an informed choice regarding interventions, (2) making a recommendation to limit interventions, (3) stating a plan to limit interventions, (4) explicitly not offering interventions, and (5) not mentioning interventions. In rare cases of intractable conflict, clinicians reported using an option of last resort: (6) invoking an institutional process to limit interventions. Respondents reported challenges with limiting interventions via the recommended approaches of shared decision-making (approaches 1-3) and institutional processes (approach 6), which sometimes discouraged the use of these approaches. While respondents recounted successfully using alternate approaches (approaches 3-5), they described interclinician and interhospital practice variation, as well as ethical and practical uncertainties.

**Conclusions and Relevance:**

In this qualitative study, clinicians reported substantial challenges using recommended approaches to limit potentially nonbeneficial life-prolonging interventions. Some clinicians reported using alternate approaches that are not supported in professional society policy statements.

## Key Points

**Question:**

What decision-making approaches do clinicians use to limit potentially nonbeneficial life-prolonging interventions?

**Findings:**

In this qualitative study of 101 clinician interviews conducted at 3 academic medical centers, respondents reported facing challenges limiting interventions using the approaches recommended in professional society policy statements (ie, shared decision-making, institutional processes addressing disagreement with patients or surrogates). Respondents described alternate approaches (eg, stating a plan to limit interventions, explicitly not offering interventions, not mentioning interventions) in which clinicians limited interventions without a shared decision or institutional process.

**Meaning:**

Clinicians face challenges in limiting potentially nonbeneficial life-prolonging interventions via recommended approaches, which may lead them to resort to alternate approaches.

## Teaser

This qualitative study describes the types of decision-making approaches clinicians reported using to limit the use of potentially nonbenefical life-prolonging interventions.

## Introduction

US hospitals, particularly academic medical centers, exhibit a default tendency toward life prolongation.^1,2,3^ Life-prolonging interventions include life-sustaining treatments (eg, mechanical ventilation, vasopressors), transfer to higher levels of care (eg, intensive care unit [ICU]), and invasive procedures (eg, hemodialysis catheter placement, bronchoscopy, extracorporeal membrane oxygenation [ECMO] cannulation). Life-prolonging interventions are often initiated, continued, and escalated by default until clinicians become concerned interventions are potentially nonbeneficial^1,3,4^—that is, clinicians believe interventions are unlikely to improve patient outcomes or quality of life and may cause harm.^5^ Because the default tendency toward life prolongation is generated and reinforced by systems-level factors,^1,2,3^ clinicians may unintentionally reach the point of providing potentially nonbeneficial life-prolonging interventions.

Concerns about the default use of life-prolonging interventions have been raised in medical and lay press,^6,7,8^ especially for patients with chronic critical illness or nearing the end of life.^9^ In addition to direct patient harm from unnecessary or unwanted interventions,^10^ other harms include financial toxicity to patients and families,^11^ increased emotional distress for the bereaved,^10^ increased health care expenditures,^12^ poor allocation of hospital resources,^13^ and clinician moral distress and burnout.^14,15^ Yet clinicians frequently report difficulties in limiting potentially nonbeneficial life-prolonging interventions.^16^ In resource-rich environments like US academic medical centers, surveyed clinicians report that up to one-tenth of critically ill patients receive life-prolonging interventions that should not be provided.^12,17,18^

Policy statements published by professional critical care societies recommend 2 approaches for limiting (ie, withholding or withdrawing) potentially nonbeneficial interventions: (1) achieving a shared decision-making with patients and/or surrogates,^19^ or (2) institutional processes that addresses disagreement with patients and/or surrogates (eg, medical futility or unilateral do-not-resuscitate policies).^20,21^ However, in practice, both approaches are inconsistently used, sometimes ineffective, and may be insufficient to counter the default tendency toward life prolongation.^22,23,24,25,26,27^ Clinicians may also use alternate approaches: They may limit life-prolonging interventions without a shared decision or an institutional process, such as when a clinician declares that a patient is not a candidate for an intervention or does not offer it.^28,29,30,31^

It is critical to understand how decisions to limit life-prolonging interventions are made in practice, especially if clinicians encounter challenges using recommended approaches. We conducted a multi-institutional qualitative interview study that aimed to describe the full range of decision-making approaches clinicians use to limit potentially nonbeneficial interventions for patients in medical wards or ICUs. By synthesizing clinicians’ reports of decision-making approaches used on the ground, we illuminate tensions between ethical ideals articulated in policy statements and clinician perceptions. This offers suggestions to inform clinical practice, ethical inquiry, empirical research, and the design of policies and guidelines.

## Methods

### Study Design

The qualitative study draws from a larger multi-institutional ethnographic project conducted at 3 tertiary academic medical centers on the US West Coast. Sites were purposively selected to reflect variation in end-of-life treatment intensity (one low-, medium-, and high-intensity site), as measured by the Dartmouth Atlas of Health Care.^32^ Between February 2018 and June 2022, we conducted interviews at these centers to explore institutional culture and decision-making practices near the end of life. This study was approved by the University of California, San Francisco institutional review board and adhered to the Standards for Reporting Qualitative Research (SRQR) reporting guideline.

### Data Collection

We recruited clinician respondents through group email solicitations, individual interview requests, and snowball sampling. We purposively sampled physicians from multiple services (emergency medicine, hospital medicine, critical care, geriatrics, and palliative care) along with nonphysician clinicians (eg, advanced practice clinicians, nurses, and social workers) likely to observe physician decision-making approaches, ensuring triangulation in reported approaches. We collected self-reported demographic data including age, years of practice, professional role, and gender because we sought diversity in respondents.

A hospitalist and sociologist (E.W.D.) conducted semistructured in-depth interviews using an interview guide^2^ examining decision-making regarding potentially nonbeneficial life-prolonging interventions. Interviews were conducted in person, with written informed consent, until the COVID-19 pandemic; videoconferencing, with verbal informed consent, was used thereafter. All interviews were audio-recorded, transcribed, and deidentified for qualitative data analysis.

### Initial Thematic Analysis

Initial analysis of the interview transcripts was conducted between January 2019 and December 2022. Several research team members (including E.W.D. and J.N.B.) conducted line-by-line thematic coding on a subset of interviews to develop a preliminary codebook. Code definitions were refined through team-based discussion. Next, several team members (including L.M.P. and J.N.B.) double-coded 20% of the entire dataset, adjudicating all code applications to consensus and finalizing the codebook. Two team members (including L.M.P.) used the final codebook to code the remainder of the dataset using ATLAS.ti software, version 24.2.0 (ATLAS.ti Scientific Software Development).

### Development of Framework

Secondary analysis of the interview transcripts was conducted between August 2023 and May 2025. Using the applied codes, we extracted all excerpts in which the respondent reported an approach used by a physician to limit a life-prolonging intervention. We analyzed these descriptions to develop a preliminary conceptual framework of clinician-reported approaches to limiting life-prolonging interventions. Drawing on clinical and bioethics literature, we began with a deductive framework (eAppendix in Supplement 1) that provided initial language and concepts to categorize reported approaches. Four investigators (J.N.B., S.W.G., A.A.N., and H.O.C.) independently reviewed and categorized each excerpt. Each excerpt was discussed in team meetings using constant comparative methods to revise the framework iteratively.^33^ Disconfirming cases helped challenge and revise preliminary categories from the literature until all reported approaches could be accounted for.^34^ To represent the framework, all identified approaches were visually organized and summarized in a figure. A core team (J.N.B., S.W.G., and J.K.A.) drafted a finalized framework, which was refined and validated by additional team members (A.A.N., H.O.C., J.A.B., J.M.K., and E.W.D.) with diverse backgrounds in hospital medicine, geriatrics, palliative care, critical care, procedural subspecialties, and bioethics for its consistency with interview data and interpretability in light of existing literature (eAppendix in Supplement 1).

### Analysis of Recommended and Alternate Approaches

We categorized each approach as a recommended approach or an alternate approach based on the critical care society policy statements that address decision-making regarding life-prolonging interventions.^19,20,21^ We recognized an approach as a recommended approach if it relied (1) on shared decision-making^19^ or (2) on an institutional process to limit an intervention.^20,21^ To verify the latter, we collected hospital policy documents describing institutional processes at each site.

We aimed to understand clinician perspectives regarding when and why they might deviate from recommended approaches. Therefore, we conducted a directed thematic analysis to identify challenges clinicians encountered using recommended approaches. Since alternate approaches are not clearly described in professional society policy statements, we used inductive thematic analysis to explore clinicians’ general perspectives regarding these approaches as they emerged in interviews.

## Results

We recruited 101 clinician respondents with approximately equal representation from each site (hospital A: 34, B: 35, C: 32), whose demographics are reported in Table 1. Our sample included 53 attending physicians (52%), 16 trainee physicians (16%), 6 advanced practice clinicians (6%), 21 nurses (21%), 3 chaplains (3%), and 2 social workers (2%), with approximately equal proportion of women (59 [58%]) and men (42 [42%]) and a wide range of ages (mean, 42 [range, 27-74] years) and experience (mean, 14 [range, 1-52] years).

**Table 1.** Respondent Demographics

Clinician demographics	All interviews, No. (%) (n = 101)
Profession	
Physician	69 (68)
By professional level	
Attending	53 (52)
Fellow	10 (10)
Resident	6 (6)
By specialty	
Emergency medicine	10 (10)
Ethics	5 (5)
Geriatrics	3 (3)
Critical care	28 (28)
Internal medicine	19 (19)
Palliative care	7 (7)
APC	6 (6)
ICU nurse	21 (21)
Chaplain	3 (3)
Social worker	2 (2)
Gender	
Men	42 (42)
Women	59 (58)
Age, mean (range), y	42 (27-74)
Years of experience, mean (range)	14 (1-52)

Abbreviations: APC, advanced practice clinician; ICU, intensive care unit.

### Framework of Clinician-Reported Decision-Making Approaches

Respondents described a range of decision-making approaches physicians use to influence patients and surrogates toward limiting potentially nonbeneficial life-prolonging interventions. Based on these descriptions, we developed a conceptual framework of 6 approaches that categorized each approach as a recommended or alternate approach (Figure). While no individual respondent articulated all 6 approaches, each was represented across sites by physician and nonphysician clinicians.

**Figure.**
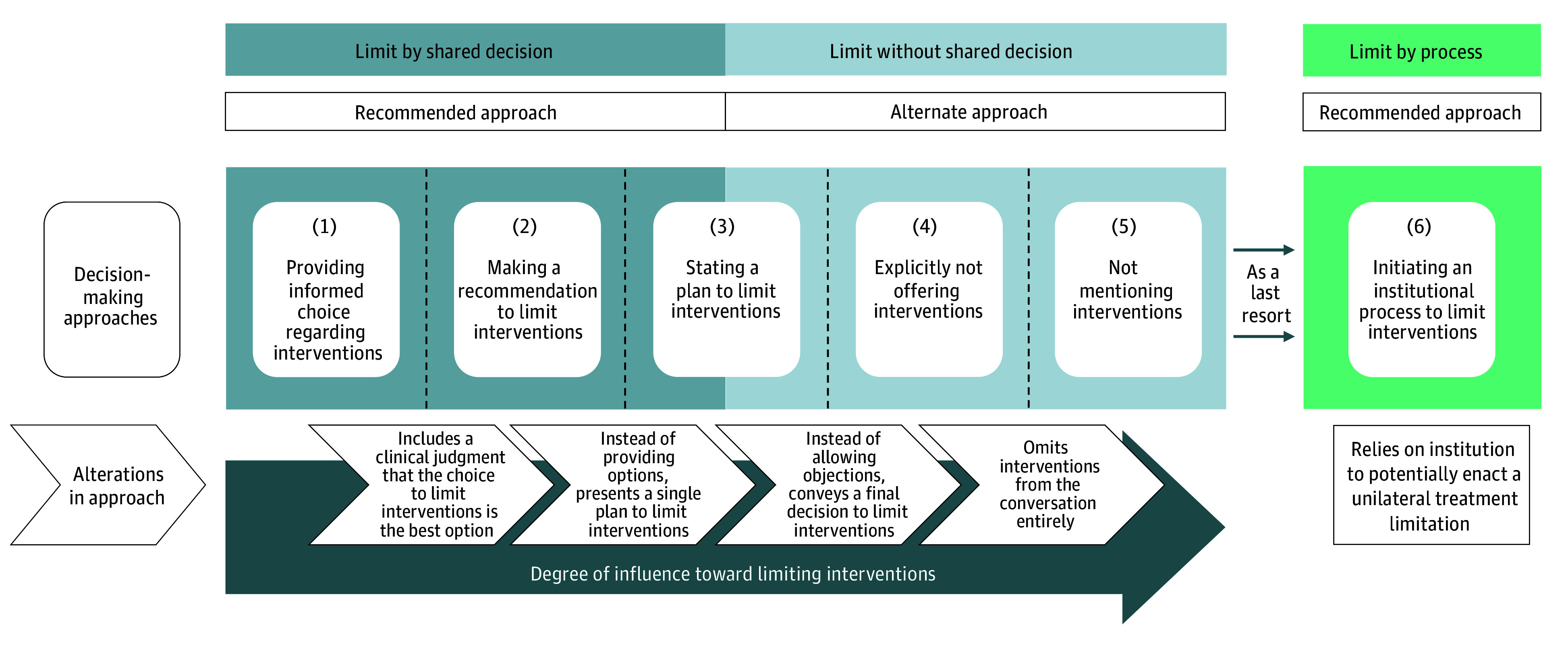
Framework of Clinician-Reported Approaches Used to Limit Life-Prolonging Interventions The 6 decision-making approaches are identified from left to right across the center of the Figure. In the boxes above, each decision-making approach is categorized as an approach that is recommended in professional society policy statements (ie, shared decision-making, institutional process addressing disagreement with patients/surrogates), or as an alternate approach. Approach 3 can be either a recommended or an alternate approach, depending on how it is enacted. For the discussion-based approaches (approaches 1-5), the alterations that physicians make to tailor the degree of influence toward limiting interventions (eg, altering what information is presented, altering how choice is offered) are described below in arrow-shaped boxes.

Respondents described approaches 1 through 5 as discussion-based approaches, in which physicians tailor the degree of influence they provide toward limiting interventions by altering the way they present information or offer choice (Figure). In contrast, respondents described approach 6 as a shift from attempting to limit interventions through discussion with the patient or surrogate to involving the institution in a policy-supported manner (Figure).

### Description of Decision-Making Approaches

Each approach is described below, with quotations shown in Table 2.

**Table 2.** Decision-Making Approaches Reported by Clinician Respondents

Approach	Defining element	Numbered quotation
(1) Providing an informed choice regarding interventions	Physician presents a set of options, with at least 1 option to limit life-prolonging interventions.	Q1: “I say, ‘These are the options on the table,’...Wanting to be somewhat realistic so that the individual can make a more informed decision…[knowing] what those interventions are, and what they could expect to see...What does it look like to be on a ventilator? What does it look like to have a central line? What does it look like to have CPR in progress?…Sometimes people have one understanding that may not be matched by the reality of those situations.” Emergency Medicine Physician 1
	Physician contextualizes options in terms that allow the patient or surrogate to make an informed choice (eg, the expected quality of life, the probability of survival to hospital discharge, etc).	Q2: “So those providers really explain that even if they are going to offer that treatment…they do not believe that it would further the patient’s life outside of the hospital or…change the final outcome of this patient’s end of life. It might change how they get there and what they endure until that happens, but it won’t change the overall prognosis or the outcome. And in many patients, in fact, it can be worse.” Nurse 1
	The provided information may guide the patient or surrogate to limit interventions.	Q3: “[I try to] open the door for them not to choose [life-prolonging interventions] and not be afraid of it.” Hospitalist 1
	This approach was frequently contrasted with offering interventions without contextualized information.	Q4: “The attending offered a tracheostomy…The attending didn’t really have a discussion with the family about what his life would look like afterwards…Very often, [interventions are offered] without the context of quality of life and values and things like that.” Palliative Care Physician 1
(2) Making a recommendation to limit interventions	Physician directly guides toward limiting interventions via a professional recommendation.	Q5: “Really thinking through the decision with [the family members]…Explaining to them what feeding tubes can and can’t do…in a way that that particular family can understand, and usually it’s giving a recommendation against a feeding tube and supporting their emotions.” Palliative Care Physician 2
	Physician explicitly recommends limiting interventions as the preferred option.	Q6: “I want [family members] to be aware of all the options…I do offer my opinion…A lot of people feel they’re giving up on family members when they don’t do absolutely everything for them…I try to take the burden off them and say that I don’t recommend doing some of these things.” Intensivist 1
	Physicians may convey the recommendation using alternate language.	Q7: “I feel more comfortable now [making recommendations] because I have had more experience to see how these things go. I frame it [as], ‘I don’t know if this would help you, but this is what I would do with my mom or dad.’” Emergency Medicine Physician 2
	The patient or surrogate may decline the physician’s recommendation.	Q8: “I don’t leave it like, ‘Here are options—just decide.’ I would say, ‘Given your mother or father...the severity of their illness, I think...They might go through a lot more pain, and it might be for a very small chance of survival at probably a low-functional status, so I wouldn’t recommend transfer to ICU.’ I do leave it up to them...to actually make the final decision.” Hospitalist 2
(3) Stating a plan to limit interventions	Physician states a plan to limit interventions.	Q9: “There is [one physician] ...He lays it out there...He says, ‘I am not going to pound on [the patient’s chest], I’m not going to break ribs, I’m not going to do that...It would only be cruelty. I’m just not willing to do that.’ He’ll say to me outside the room, ‘So, I’m willing to be confident...with my delivery. And I am very happy to back down if [the family members] go bonkers over this.’” Social Worker 1
	Physician proceeds with the plan if there is no objection.	Q10: “I might say, ‘If his heart were to stop, we have the whole hospital come running, and they try chest compressions. I don’t think [that] would be reasonable.’ And then I pause and say, ‘There’s something I would put in the chart; it’s called DNR.’… [This approach] is a stronger thing; it’s not just, ‘This is what I would suggest.’ I think even [a recommendation] is sometimes too hard for families because it’s their decision again. And if I assent them to putting it in the chart, I can say that I talked to them about it, and they were okay with it.” Intensivist 2
	Physicians differed in the degree to which they offered explicit choice to defer to physician judgment or object to the plan.	Q11: “Because some families are paralyzed by…the burden of decision…I would tell them, ‘Okay, it’s not you making the decision, it’s me as the treating physician making this decision. You’ve given me the information about what he or she loved doing, what their quality of life was, what they had told you about what they would want if they were in a similar situation. Now it’s up to me to as the physician do what I think this patient would tell me to do.’” Intensivist 3
	Physician provides guidance by reducing a decision to a single option.	Q12: “[Family members] are looking for the burden to come off their shoulders…If somebody else is telling them ‘We’re going to do this,’ then their decision-making is gone, and they can focus on their loved one without having to deal with making any sort of stressful decisions at the end.” Nurse 2
(4) Explicitly not offering interventions	Physician conveys a final decision to limit interventions.	Q13: “I would say to the family, ‘I think Mrs [X]’s kidneys have shut down, and they’re failing. I think that it’s not an appropriate use doing dialysis in this situation. I think she’s telling us that she’s dying. And so, I am not going to offer dialysis in this situation.’” Intensivist 4
	This approach is not responsive to objections from the patient or surrogate.	Q14: “We told the family absolutely not [to CPR]. The family objected. We said we would not offer it. We felt we had an obligation only to offer therapies that had some potential of being effective. In this case, we were firm in our belief that it wouldn’t.” Intensivist 5
	Physician provides guidance by explicitly removing the possibility of interventions.	Q15: “I see the dichotomy…because you’re like, ‘I’m intubating a patient, when I know that this is not going to change the ultimate outcome.’ But a little bit is like getting the family some time to understand and getting them to that point to know to let go. Some of our faculty are like, ‘Yes, I would just talk to them up front and not offer mechanical ventilation.’” Emergency Medicine Physician 3
(5) Silently withholding interventions	The physician omits an intervention from discussion entirely.	Q16: “I don’t feel like I need to present every single option…I don’t necessarily present everything we can do.” Critical Care Fellow 1
	In some circumstances, the physician does not consult the team responsible for the intervention.	Q17: “For dialysis, I have flat out told patients and families, ‘That won’t benefit you’…But it’s interesting, if [nephrology] is involved, it just depends on the attending [nephrologist] whether it’s offered...I have had patients where, knowing the nephrologist who was on, I have not called [nephrology] because I did not want that patient to end up on dialysis.” Hospitalist 3
	Physician provides guidance by avoiding the introduction of additional interventions.	Q18: “Sometimes, I give a little bit less information if I think [family members] are going to grab on to something that has a 0.0005% chance of happening. So, I guess in that sense, sometimes I don’t offer ICU-level care.” Palliative Care Physician 3
(6) Invoking an institutional process to limit interventions	The institutional process limits interventions over the objections of the patient or surrogate.	Q19: “She...had an in-hospital cardiac arrest...[She] started to develop renal failure…It [had] been 3 months in the ICU. Her husband was adamant about doing...every possible intervention, even if it was causing her discomfort. We did a unilateral ‘no dialysis’ [and] gave them [the] 7-day notification...We ended up starting dialysis but said we would only continue it for 7 days...Husband didn’t find a facility, [so we] took her off dialysis, and she passed away.” Hospitalist 4
	The process is supported by hospital policy, structures, and personnel in authority positions.	Q20: “We have an ethics policy where, if there is extreme conflict between the doctor’s recommendations and the family’s wishes, we can bring it to the Ethics Committee. We have a mechanism where if the Ethics Committee decides in the doctor’s favor, there’s a hospital policy where it can overrule the family.” Intensivist 6
	Described as an option of last resort.	Q21: “I really try to reserve unilateral decision-making as a last resort. As physicians, we’re making the final sacrifice to do what we think is best for the patient.” Hospitalist 5

Abbreviations: CPR, cardiopulmonary resuscitation; DNR, do not resuscitate; ICU, intensive care unit.

1. **Providing an informed choice regarding interventions:** The physician presents multiple options to the patient or surrogate, including at least 1 that limits life-prolonging interventions (Q1). The physician contextualizes these options to support an informed choice (eg, by describing expected quality of life, probability of survival to hospital discharge, etc) (Q2). Respondents felt this framing could influence patients and surrogates to limit interventions (Q3). Some respondents contrasted this approach with offering interventions without any guidance or intention of limiting interventions (Q4).
2. **Making a recommendation to limit interventions:** The physician presents multiple options to the patient or surrogate and conveys a professional recommendation to limit interventions (Q5). Respondents provided examples of explicit recommendations (Q6) and discussed other ways recommendations could be conveyed (Q7). Respondents noted that the physician allowed the patient or surrogate to choose options differing from the recommendation (Q8).
3. **Stating a plan to limit interventions:** The physician states a plan to limit interventions in the presence of known alternatives (Q9) and proceeds unless the patient or surrogate dissents (Q10). Physicians differed in the degree to which they explicitly offered a choice to defer to physician judgment or object to the plan (Q9-Q11). Respondents felt this approach guided the patient or surrogate toward limiting interventions by easing their decision-making burden (Q10-Q12).
4. **Explicitly not offering interventions:** The physician states a final decision to limit interventions without providing a choice to the patient or surrogate (Q13). The decision not to offer an intervention is not responsive to objections from the patient or surrogate (Q14). Respondents explained that this approach guided the patient or surrogate by removing options from consideration (Q15).
5. **Silently withholding interventions:** The physician omits mention of a life-prolonging intervention, thereby offering neither information nor choice (Q16). Some physicians described how this could be accomplished by not consulting physicians who might offer the intervention (Q17). Respondents saw this approach as a way to avoid introducing new interventions as options (Q18).
6. **Initiating an institutional process to limit interventions:** The physician activates an institutional policy to limit interventions over the objections of the patient or surrogate (Q22). Most physicians described a process that involved other hospital personnel in positions of authority (eg, ethics consultants, risk management, the chief medical officer) (Q23). It was consistently described as an option of last resort that was rarely used (Q24).

### Recommended Approach: Shared Decision-Making

In approaches 1 through 3 (providing an informed choice regarding interventions, making a recommendation to limit interventions, stating a plan to limit interventions), the physician guides the patient or surrogate toward limiting interventions but proceeds only if a shared decision is reached, conforming to recommendations in professional society policy statements.

Respondents identified challenges to limiting interventions using shared decision-making (Table 3). Respondents described how patients and surrogates experienced extreme difficulties in making potentially life-ending decisions (Q22), resulting in time-consuming and distressing decision-making experiences for all parties (Q23). During prolonged decision-making, respondents expressed alarm about unnecessary patient suffering or harm (Q24) and moral distress for the clinical team (Q25). Some respondents acknowledged that such challenges influenced them to avoid shared decision-making in certain situations (Q26).

**Table 3.** Respondent Perspectives on Challenges Encountered With Recommended Approaches

Approach	Challenge	Quotation
Limiting interventions by shared decision	Patients and surrogates face profound difficulties in making potentially life-ending decisions.	Q22: “[The son] could not make the decision to make his mother DNR. It was a horrible situation because the daughters were like, ‘Oh, she’s a fighter. Please do everything.’ And then when they saw the chest compressions being done and then, eventually, she was pronounced, they were like, ‘I hope she didn’t suffer.’…We had so many discussions with them that she’s not going to survive this.” Intensivist 6
	Achieving a shared decision can be time consuming and distressing for all parties.	Q23: “They do a lot of family meetings to discuss goals of care. I feel like those are usually good stepping tools towards making decisions. But sometimes…the process is just so delayed and drawn out that you can have 10 family meetings when we know what was supposed to happen at the first one…Maybe it is just that it takes patients and the person’s disease to dry out for a couple of weeks for the family to finally realize, ‘Okay, there’s really no hope.’ But it’s just sad when [patients] have to suffer.” Nurse 3
	During prolonged decision-making, clinicians express alarm about patient suffering.	Q24: “They did not realize that it was 5 days later—because they were still trying to resolve with the family and trying to help them get toward comfort care and stopping dialysis. In that case, we sat and had a conversation about where [are we] not seeing eye to eye so to speak, and what was distressing them so much. And part of it was that in those 5 days after I had given my opinion, she had been up to 1 week in restraints, [...] being tied down and suffering.” Hospitalist 1
	During prolonged decision-making, clinicians experience moral distress.	Q25: “It’s morally distressing for us. I think a lot of us...We talk a lot about it with each other, but a lot of times we’re morally distressed because we’re either inflicting pain on the patient…but the family is saying, ‘Keep fighting. We love you,’ and this and that. I think sometimes we feel like we’re doing more harm than good because we have to do this.” Nurse 4
	Given the challenges associated with achieving a shared decision, physicians were sometimes hesitant to use this approach.	Q26: “For some surrogates, it’s such a burdensome process to be a surrogate—you have to make the decisions, and the burden falls on you. I do feel like for us it’s easier to have that burden on us than for family members to have that burden, so that feels appropriate, and, as an objective person, it feels easier for me to make tough choices on their behalf.” Hospitalist 6
Limiting interventions by institutional process	Limiting by institutional process can lead to traumatic experiences for patients and surrogates.	Q27: “The unilateral decision of providers to do what they think is ethical or appropriate is always an option, but it’s not an option I like to use—it leaves a wake of resentment and suffering that may last the entire life of the survivors. I don’t like that as a principle.” Palliative Care Physician 2
	Limiting by institutional process can lead to larger negative consequences (ie, litigation, negative press).	Q28: “[Using an institutional process to limit interventions] can lead to court orders to continue. That situation can lead to families trying to talk to the press about you…So, why would you, as a physician, choose to do some of those things? I mean, if you could just do your week and move on, right? So, I think it creates a lot of conflict…to do something unilaterally.” Intensivist 7
	The entire clinical team may not commit to limiting by institutional process.	Q29: “He had 3 ethics counsels, which is a lot for one patient. I was like, ‘Well, he has a unilateral DNR from ethics, but depending on what physician you have, it may or may not be followed’…If we didn’t want to code him, we wouldn’t have had to, and we would have been protected. But it’s just the path of least resistance [to perform CPR].” Nurse 5
	Given the challenges associated with using an institutional process, physicians were sometimes hesitant to use this approach.	Q30: “You may or may not do it [use an institutional mechanism to limit interventions] depending on how much you want to deal with it, on one hand, and how much you think your patient is going to die anyway in the next few days. Maybe you’re not going to invest all this time and effort into getting ethics and all this stuff involved.” Intensivist 8

Abbreviations: CPR, cardiopulmonary resuscitation; DNR, do not resuscitate.

### Recommended Approach: Institutional Processes

Approach 6 (initiating an institutional process to limit interventions) also conforms to recommendations in professional society policy statements. Respondents identified challenges with using this approach (Table 3). Respondents felt that, while this approach was sometimes necessary, it was likely to worsen clinicians’ relationship with the patient or surrogate, causing resentment or suffering (Q27). Respondents feared downstream negative consequences such as legal action or media scrutiny (Q28). Respondents felt that the success of this approach depended on a unified team and administrative support, which was not always present (Q29). Some respondents acknowledged that such challenges deterred them from using institutional processes to limit interventions (Q30).

### Alternate Approaches

In approaches 4 and 5 (explicitly not offering interventions, not mentioning interventions), the physician limits interventions without a shared decision or an institutional process. In most descriptions of approach 3 (stating a plan to limit interventions), the patient or surrogate is not offered an opportunity to object to the stated plan, and thus the physician limits interventions without a shared decision. These approaches are not recommended in professional society policy statements.

While respondents recognized that these alternate approaches were sometimes used (Q30) (Table 4), they described substantial practice variation in the use of alternate approaches among individuals (Q30 and Q31) and across institutions (Q32). Respondents felt that physicians differed in their ethical beliefs about alternate approaches (Q33), with some questioning (Q34) and others endorsing (Q35) the ethical permissibility of alternate approaches. Respondents used various rationales to justify alternate approaches, such as lack of impact on patient outcome, potential for harm, medical ineffectiveness, or medical futility (Q36). Respondents also described specific uncertainties regarding the permissibility of alternate approaches, depending on the nature of the intervention (Q36) or the physician’s specialty or role (Q37). These ethical uncertainties were closely intertwined with practical uncertainties, including how such approaches might be received by patients and surrogates or consulting teams and the potential for negative downstream consequences (Q36 and Q37).

**Table 4.** Respondent Perspectives Regarding Alternate Approaches

Category	Perspective	Numbered quotation
Physician use of alternate approaches	Some physicians limit life-prolonging interventions without a shared decision and without an institutional process.	Q30: “[Some clinicians] feel it’s their prerogative as experts to make decisions [to limit life-prolonging interventions] and to inform family members rather than assenting or consenting them. If there’s a very strong case for why something shouldn’t happen, they use their medical knowledge and interpretation of what’s happening and make those decisions, and they don’t ask anybody. But those are very specific individuals who I know feel comfortable doing that.” Palliative Care Physician 3
	There is interclinician variation in the use alternate approaches.	Q31: “[Willingness to not offer dialysis] is totally variable. I think some of it is dependent upon the experience of the attending…the farther you are from your training, the more likely you are to make the decision of not offering dialysis.” Intensivist 9
	There is interhospital variation in the use of alternate approaches.	Q32: “[Not offering interventions] was more part of the culture there [at another hospital] than was shared decision-making…our job was first do no harm, and [offering interventions] was thought of as me potentially causing harm when I certainly can’t cause you good. But here [at my current hospital], I think there’s also a sense that we’re treating the family, and so maybe by doing these procedures, I’m actually doing good because I’m treating the whole system around [the patient].” Intensivist 10
Ethical questions surrounding the use of alternate approaches	Physicians differ in their beliefs regarding the ethical permissibility of alternate approaches.	Q33: “Certain attendings wouldn’t offer dialysis, others would…based on their general thought and beliefs [which] can change from attending to attending.” Hospitalist 6
	Some respondents question the ethical permissibility of alternate approaches.	Q34: “I run into situations where a tracheostomy and life in the facilities are completely appropriate and in line with the patient’s goals. And that procedure was going to be withheld. Because it was felt that there’s no way a person would want to live this way. We made our assumptions about what is an acceptable quality of life to a person…We’re on shaky ground to make blanket decisions about whether or not a treatment should be offered.” Palliative Care Physician 4
	Some respondents explicitly endorse the ethical permissibility of alternate approaches.	Q35: “I feel pretty comfortable saying, ‘We’re not going to do CPR because it’s just going to prolong death’…I don’t know if all of my colleagues are comfortable saying that…But I tell the house staff that you don’t have to offer things to people that are not going to be helpful. You’re not obligated to Zosyn someone if you don’t think they have pneumonia just because they want Zosyn. CPR is the same thing.” Intensivist 11
	Respondents used various rationales to justify alternate approaches.	Q36: “I do not feel bound to offer treatments that I think are not going to change the outcome. So, in situations where I don’t think it’s going to change the outcome, then I won’t offer treatments.” Intensivist 12
	There is uncertainty regarding the use of alternate approaches depending on the intervention.	Q37: “If their patient dies without that discussion [about a life-prolonging intervention] explicitly being had, are they going to regret that? Are they going to feel that there was any dishonesty or lack of complete sharing of information?…Whereas with ECMO, that’s less of a standard where someone’s going to say, ‘Well, was ECMO talked about?’ Whereas if someone says, ‘Well, did they talk about dialysis,’ and it wasn’t [discussed], then someone will say, ‘Well, why didn’t they?’” Intensivist 13
	There is uncertainty regarding the use of alternate approaches, depending on physician specialties.	Q38: “Surgeons [don’t offer interventions] all the time: ‘No, I’m not going to operate on you.’ It doesn’t mean you can’t get a second opinion…Dialysis is a perfect example where I say ‘I don’t think dialysis is a good idea. I don’t think it’s going to reach these goals.’ [The family says,] ‘Can we talk to the kidney doctor?’ Of course, right? You can’t just say I’m the dictator...You have to allow people to talk to someone else. And so, then [the] kidney doctor shows up and says, ‘Of course we can do dialysis.’” Intensivist 14

Abbreviations: CPR, cardiopulmonary resuscitation; ECMO, extracorporeal membrane oxygenation.

## Discussion

This multi-institutional qualitative interview study with clinicians across 3 academic medical centers characterizes the full range of approaches physicians use to limit potentially nonbeneficial life-prolonging interventions (Figure). The 6 approaches identified in this study were reported across hospitals that varied in geographic location and end-of-life treatment intensity, suggesting that clinicians across institutions navigate similar decisional pressures, likely shaped by a shared default tendency toward life prolongation in the US.^1,2,3,4^ Respondents at all sites perceived a need to mitigate the harms associated with this default by influencing the patient or surrogate toward limiting potentially nonbeneficial interventions.

This study provides 2 central contributions to the clinical and bioethics literature. First, it introduces a framework that organizes the decision-making approaches physicians use to influence a patient or surrogate to limit life-prolonging interventions, offering a structured set of approaches and shared language for coordinating sensitive conversations. The framework highlights how deliberations about life-prolonging interventions do not occur in a neutral space but rather in the context of a default tendency to initiate, continue, and escalate life-prolonging interventions. In this study, respondents described how physicians intentionally disrupt this default pathway by using decision-making approaches that influence patients and surrogates toward limiting potentially nonbeneficial life-prolonging interventions.

Second, this study provides evidence that physicians sometimes limit life-prolonging interventions without a shared decision or an institutional process. Respondents described how physicians resorted to these alternate approaches when life-prolonging interventions were highly unlikely to provide net benefit, but recommended decision-making approaches appeared burdensome or ineffective. In this way, alternate approaches could be viewed as pragmatic workarounds.^35,36^ In the sociological literature, workarounds are defined as “shortcuts or fixes for when work rules don’t match work realities.”^36^ Physicians resorted to alternate approaches when there were no other routes for addressing the problem of potentially nonbeneficial life-prolonging interventions. Recognizing alternate approaches as pragmatic workarounds underscores the limitations of professional society policy statements in a health care system that tends to intervene by default.

Respondent descriptions of the challenges associated with recommended approaches (Table 3) offer suggestions for ways forward. Respondents described shared decision-making as ineffective when patients or surrogates struggled to make potentially life-ending decisions, in agreement with prior literature.^37,38,39,40^ In these situations, respondents framed alternate approaches as strategies to reduce the emotional burden of a decision by reshaping choice architecture or pre-empting a difficult choice altogether.^41,42,43^ Similarly, respondents described institutional processes as burdensome, costly, and risky—especially when clinical team or administrative consensus could not be achieved, also aligning with prior literature.^22,26^ By incorporating clinicians’ experiences of these constraints, hospital-level interventions, hospital policies, and professional society statements might be designed to better support ethical decision-making.

Examining the conceptual boundaries between the recommended and alternate decision-making approaches is also instructive. First, policy statements recommend not offering or not mentioning interventions only in situations of physiologic futility when interventions cannot meet their intended goal (eg, providing antifungals for a myocardial infarction).^20,21^ In the present study, clinicians reported not offering or not mentioning interventions that had at least some chance of prolonging life, suggesting that physiologic futility may be too rigorous to address everyday clinical challenges. While policy statements allow exceptions in time-pressured situations,^20,21^ some clinicians reported not offering or not mentioning interventions without apparent time pressure.

Second, policy statements and other literature describe informed assent and nondissent as forms of shared decision-making only if patients and surrogates are informed of other options, understand that they are deferring to physician judgment, and can object to the plan of care.^19,44,45,46,47^ In the present study, most reports of stating a plan did not consistently meet these requirements, suggesting that that these requirements are challenging to implement for clinicians facing the high-stakes, emotionally laden discussions that engender use of this approach.

When physicians limit life-prolonging interventions without a shared decision, they risk imposing physician values and biases on vulnerable patients for preference-sensitive, high-stakes decisions.^20,48^ While some patients and surrogates prefer clinician-directed approaches, others prefer to direct decision-making themselves,^19,49,50^ inconsistent with the alternate approaches identified. This study points to a need for additional ethical inquiry and multidisciplinary professional consensus around when, if ever, alternate approaches are permissible.^28,29^ This need is especially pronounced given the confusion noted by respondents around the ethical permissibility of alternate approaches, particularly concerning the nature of the intervention (eg, not offering mechanical ventilation vs ECMO) and the role and specialty of the physician (eg, consulting surgeon vs hospitalist). Although it is well accepted that surgeons and other proceduralists can not offer interventions, it is unclear whether similar professional discretion should be extended to hospitalists, intensivists, and other medical subspecialists.^28,29^ At present, the alternate approaches described in this study are not supported by professional policy statements, and there is no explicit consensus regarding their ethical permissibility.

### Limitations

This study has several limitations. Our analysis is based on clinician reports rather than observed behavior, which limits our ability to understand clinical context and may introduce reporting bias. However, we triangulated findings across institutions and a varied sample of clinicians to strengthen the validity of our framework. Further work, likely in the form of ethnographic observation, is needed to better understand how and when clinicians resort to alternate approaches. Additionally, semistructured qualitative interviews cannot support a comparative analysis among hospitals or clinicians, although some respondents reported interhospital or interclinician variation.

## Conclusions

In this multi-institutional qualitative study, respondents reported facing challenges limiting interventions using the approaches recommended in professional society policy statements (ie, shared decision-making and institutional processes addressing disagreement with patients and surrogates). Use of alternate approaches (eg, stating a plan to limit interventions, explicitly not offering interventions, and not mentioning interventions) can be viewed as pragmatic attempts to navigate decision-making challenges surrounding life-prolonging interventions. Strategies are needed to help clinicians balance the ethical ideals articulated in professional societal policy statements with the potential harms imposed by the default tendency toward life prolongation in US hospitals.

## Notes

Supplement 1. **eAppendix.** Literature-Based Framework of Decision-Making Approaches Used by Clinicians to Limit Potentially Nonbeneficial Life-Prolonging Interventions
eReferences

Supplement 2.
Data Sharing Statement
