# Supplementary material for: Decision-Making Approaches Used to Limit Potentially Nonbeneficial Life-Prolonging Interventions
Source: JAMA Netw Open. 2026 Feb 20;9(2):e2560260. doi: 10.1001/jamanetworkopen.2025.60260 (PMC12924098; doi:10.1001/jamanetworkopen.2025.60260)
Supplement: Supplement 1. — eAppendix. Literature-Based Framework of Decision-Making Approaches Used by Clinicians to Limit Potentially Nonbeneficial Life-Prolonging Interventions eReferences [file jamanetwopen-e2560260-s001.pdf]

Supplemental Online Content

Batten JN, Goitiandia SW, Axelrod JK, et al. Decision-making approaches used to limit potentially nonbeneficial life-prolonging interventions. *JAMA Netw Open*. 2026;9(2):e2560260. doi:10.1001/jamanetworkopen.2025.60260

eAppendix. Literature-Based Framework of Decision-Making Approaches Used by Clinicians to Limit Potentially Nonbeneficial Life-Prolonging Interventions
eReferences

This supplemental material has been provided by the authors to give readers additional information about their work.

© 2026 Batten JN et al. JAMA Network Open.

eAppendix: Literature-Based Framework of Decision-Making Approaches Used by Clinicians to Limit Potentially Nonbeneficial Life-Prolonging Interventions

Our categorization of clinician reports of decision-making approaches used to limit life-prolonging interventions began with an initial framework based on references from the clinical and bioethics literature. The table below describes our initial categories of decision-making approaches along with their defining elements. The table also provides the key references and a brief description of how each reference contributed to the formation of initial categories.

Decision-Making Approach and Defining Elements	Key Literature Informing Initial Description of Approach
**Autonomy-Driven Shared Decision**<br>• Clinician discusses at least 2 options;<br>• Clinician facilitates a deliberation about the options;<br>• Clinician does not provide a recommendation;<br>• Patient/surrogate decision-maker provides informed consent;<br>• Clinical team implements the option chosen by patient/surrogate.	**Schwarze et al. 2005. You Can't Get What You Want: Innovations in End-of-Life Communication in the Intensive Care Unit.** Includes an example from a goals of care conversation of a clinician providing a choice to continue life-prolonging interventions or pursue comfort care. The clinician facilitates a discussion about the patient's wishes but does not provide a recommendation.[2]<br><br>**White et al 2010. Expanding the paradigm of the physician's role in surrogate decision-making: An empirically derived framework**. In clinician-surrogate conferences for critically ill patients, some physicians played a "facilitative role" in which they refrained from providing a recommendation but actively guided the surrogate through a decision-making process. It also notes that some physicians refused to provide a recommendation when it was requested by the surrogate.[3]<br><br>**Dzeng et al. 2015. Influence of institutional culture and policies on do-not-resuscitate decision making at the end of life.** Illustrates that clinicians who prioritize autonomy may feel compelled to offer the choice of resuscitation to all patients, regardless of whether they believed resuscitation to be clinically appropriate.[4]<br><br>**Kon et al 2017. Shared Decision Making in Intensive Care Units: An American College of Critical Care Medicine and American Thoracic Society Policy Statement.** This policy statement cites prior empirical research showing that some surrogates prefer to direct decision-making without a clinician's recommendation.[5]
**Beneficence-Driven Shared Decision**<br>• Clinician discusses at least 2 options;<br>• Clinician facilitates a deliberation about the options;<br>• Clinician provides a recommendation in favor of a treatment limitation;	**White et al 2010. Expanding the paradigm of the physician's role in surrogate decision-making: An empirically derived framework**. In clinician-surrogate conferences for critically ill patients, some physicians played a "collaborative role" in which the physician facilitated deliberation about a treatment decision and provided a recommendation to the surrogate.[3]<br><br>**Dzeng et al. 2015. Influence of institutional culture and policies on do-not-resuscitate decision making at the end of life.** Illustrates that clinicians who prioritize the patient's best

© 2026 Batten JN et al. JAMA Network Open.

• Patient/surrogate provides informed consent.<br>• Clinical team implements limitation only when patient/surrogate agrees to it.	interest appeared to be more comfortable recommending against resuscitation in situations where survival was unlikely.[4]<br><br>**Kon et al 2016. Shared Decision Making in Intensive Care Units: An American College of Critical Care Medicine and American Thoracic Society Policy Statement.** This policy statement cites prior empirical research demonstrating that some surrogates prefer to make decisions based on a clinician's recommendation.[5]
**Informed Assent / Non-Dissent**<br>• Clinician explicitly offers patient/surrogate the opportunity to defer to the physician's judgment;<br>• Clinician explains relevant information about treatment option(s) to the patient/surrogate;<br>• Clinician proposes a plan to limit interventions;<br>• Clinical team implements limitation only if there is tacit agreement or lack of dissent; full informed consent is not required.	**Kon 2010. The Shared Decision-Making Continuum.** Defines informed non-dissent, in which a clinician fully informs the patient, proposes a plan, and proceeds if the patient/surrogate does not veto the plan. The patient/surrogate must understand all pertinent information and understand that silence will be construed as tacit agreement to the plan.[6]<br><br>**Curtis 2012. The Use of Informed Assent in Withholding Cardiopulmonary Resuscitation in the ICU.** Defines informed assent, in which a clinician provides full information about expected or requested treatments, conveys a recommendation to withhold or withdraw the treatment, and offers the family the choice to defer to the clinician's judgment.[7]<br><br>**Kon et al 2016. Shared Decision Making in Intensive Care Units: An American College of Critical Care Medicine and American Thoracic Society Policy Statement.** This policy statement clarifies that informed non-dissent and informed assent, the two approaches referenced above, are different names for the same approach. It affirms that informed assent/non-dissent is a form of shared decision making.[5]
**Explicit Decision to Not Offer**<br>• Clinician explains to patient/surrogate that an intervention that will not be offered.<br>• The approach to limiting treatment is framed as a clinical decision and is not supported by a hospital policy or procedure.<br>• May include physiologic futility.	**White et al 2010. Expanding the paradigm of the physician's role in surrogate decision-making: An empirically derived framework**. In clinician-surrogate conferences for critically ill patients, one physician played a "directive role" in which cardiopulmonary resuscitation was explicitly not offered to the surrogate.[3]<br><br>**Wicclair and White 2014. Surgeons, Intensivists, and Discretion to Refuse Requested Treatments.** Highlights a discrepancy between the perceived discretion afforded to surgeons, as compared to intensivists, in withholding interventions. While surgeons are rarely challenged when they state a patient "is not a surgical candidate," intensivists are frequently challenged regarding similar decisions to not offer interventions.[8]<br><br>**Bosslet et al. 2015. An Official ATS/AACN/ACCP/ESICM/SCCM Policy Statement: Responding to Requests for Potentially Inappropriate Treatments in Intensive Care Units.** This policy statement recommends that clinicians should refuse to provide futile interventions, defined strictly as "the rare circumstance that an intervention simply cannot accomplish the intended physiologic

© 2026 Batten JN et al. JAMA Network Open.

	goal." A multi-step conflict resolution process is not recommended in these cases.[9]<br><br>**Magnus and Rizk 2016. Professional Judgment and Justice: Equal Respect for the Professional Judgment of Critical-Care Physicians.** Observes that it is very common for consulting interventionists (e.g., surgeons, anesthesiologists, interventional radiologists) to make independent judgments about whether to offer an intervention without seeking informed consent or refusal. It highlights that intensivists would normally need to invoke futility or other policy mechanism in order to refuse to offer ICU-level care.[10]<br><br>**Wong et al 2019. Care Practices for Patients with Advanced Kidney Disease Who Forgo Maintenance Dialysis.** Illustrates that nephrologists sometimes document that a patient is "not a candidate" for dialysis based on particular patient characteristics or perceived poor prognosis. Dialysis was not provided to such patients in this study.[11]
**Silent Decision to Not Offer**<br>• A decision is made to not offer an intervention; however, this is *not* discussed with the patient/surrogate.<br>• The treatment limitation is framed as a clinical decision and is not supported by a hospital policy or procedure.<br>• May include physiologic futility.	**Whitney and McCullough, 2007. Physicians' Silent Decisions: Because Patient Autonomy Does Not Always Come First.** Argues that there is a limited set of clinically significant, ethically valid "silent" decisions – that is, decisions when a physician makes a treatment decision on behalf of the patient without information the patient/surrogate.[12]<br><br>**Curtis and Burt, 2007. Point: The Ethics of Unilateral "Do Not Resuscitate" Orders: The Role of "Informed Assent."** In a discussion about categories of withholding and withdrawing life support, states that "clinicians need not discuss each treatment withheld if they do not regard it as medically indicated."[13]
**Unilateral Do Not Resuscitate Order**<br>• Clinical team, with administrative support, withholds cardiopulmonary resuscitation against patient or surrogate wishes.<br>• The process is supported by hospital policy, state law, or professional society guidelines.	**Courtwright et al. 2015. Experience with a Hospital Policy on Not Offering Cardiopulmonary Resuscitation When Believe More Harmful than Beneficial.** Retrospectively describes a unilateral DNR policy at an American hospital. The policy "provided a mechanism for not offering CPR" and required ethics committee consultation.[14]<br><br>**Robinson et al. 2017. After the DNR: Surrogates Who Persist in Requesting Cardiopulmonary Resuscitation.** Qualitatively describes the implementation challenges associated with a unilateral DNR policy at an American hospital. The policy "provided a mechanism for physicians not to offer CPR."[15]<br><br>**Putman et al. 2017. Unilateral Do Not Resuscitate Orders: Physician Attitudes and Practice.** This nationwide survey found that 20% of pulmonary critical care physicians reported performing a unilateral DNR in the previous year. The survey does not clarify whether this was supported by a hospital policy.[16]

© 2026 Batten JN et al. JAMA Network Open.

**Futility or Potentially Inappropriate Treatment Policy**<br>• Using a policy-defined process, the clinical team withholds or withdraws life-prolonging interventions against patient/surrogate objections.<br>• The process is supported by hospital policy and procedure and may be supported by state law.	**Bosslet et al. 2015. An Official ATS/AACN/ACCP/ESICM/SCCM Policy Statement: Responding to Requests for Potentially Inappropriate Treatments in Intensive Care Units.** In cases of intractable conflict with surrogates over the use of potentially inappropriate treatments, this policy statements recommends that clinicians should initiate a multi-step conflict-resolution process that may culminate in limiting life-prolonging interventions.[9]<br><br>**Kon et al. 2016. Defining Futile and Potentially Inappropriate Interventions: A Policy Statement from the Society of Critical Care Medicine Ethics Committee.** This policy statement defines futile or inappropriate ICU interventions as those that give no reasonable expectation of improving the patient's health enough for them to survive outside the acute care setting or improving the patient's neurologic function enough for them to perceive the benefits of treatment. In cases where surrogates request futile treatment and conflict cannot be resolved by shared decision-making, clinicians should initiate hospital policy to limit treatment.[17]

Additional references:
Some key references provided conceptual foundations that informed the entire initial framework.

Whitney 2003. A New Model of Medical Decisions: Exploring the Limits of Shared Decision Making. Describes a model for medical decisions in which not all decisions ought to be shared: some fall into the "zone of patient priority," others fall into the "zone of physician priority," and still others are ideally shared between physicians and patients.[18]

Whitney et al. 2004. A Typology of Shared Decision Making, Informed Consent, and Simple Consent. Discusses the relationship between shared decision making and informed consent. For discussions about life-prolonging interventions, notes that at least two alternatives would need to be discussed for a decision to be considered a shared decision in which the patient/surrogate shares priority for the decision and also provides informed consent.[19]

Whitney et al. 2008. Beyond Shared Decision Making: An Expanded Typology of Medical Decisions. Discusses clinical scenarios in which physician judges one course of clinical management as clearly optimal and recommends this course of action, and may or may not fully discuss the inferior alternative.[20]

Kon 2010. The Shared Decision-Making Continuum. Describes a range of approaches to shared decision-making, with some being more physician-driven and others being more patient-driven. Argues that physicians should only independently make value-neutral decisions (e.g., endotracheal tube size).[6]

Drolet and White 2012. Selective Paternalism. Argues for the moral necessity of acting paternalistically to support the patient's best interest in selected cases, including some scenarios ralted to the withdrawal of life-prolonging interventions.[21]

Kon et al 2016. Shared Decision Making in Intensive Care Units: An American College of Critical Care Medicine and American Thoracic Society Policy Statement. This policy statement defines major elements of shared decision making: information exchange, deliberation, and making a treatment decision.[5]

© 2026 Batten JN et al. JAMA Network Open.

eReferences

1. O'Brien BC, Harris IB, Beckman TJ, Reed DA, Cook DA. Standards for Reporting Qualitative Research: A Synthesis of Recommendations. *Acad Med*. 2014;89(9):1245. doi:10.1097/ACM.0000000000000388

2. Schwarze ML, Campbell TC, Cunningham TV, White DB, Arnold RM. You Can't Get What You Want: Innovation for End-of-Life Communication in the Intensive Care Unit. *Am J Respir Crit Care Med*. 2016;193(1):14-16. doi:10.1164/rccm.201508-1592OE

3. White DB, Malvar G, Karr J, Lo B, Curtis JR. Expanding the paradigm of the physician's role in surrogate decision-making: An empirically derived framework*. *Crit Care Med*. 2010;38(3):743. doi:10.1097/CCM.0b013e3181c58842

4. Dzeng E, Colaianni A, Roland M, et al. Influence of Institutional Culture and Policies on Do-Not-Resuscitate Decision Making at the End of Life. *JAMA Intern Med*. 2015;175(5):812-819. doi:10.1001/jamainternmed.2015.0295

5. Kon AA, Davidson JE, Morrison W, Danis M, White DB. Shared Decision Making in Intensive Care Units: An American College of Critical Care Medicine and American Thoracic Society Policy Statement. *Crit Care Med*. 2016;44(1):188-201. doi:10.1097/CCM.0000000000001396

6. Kon AA. The shared decision-making continuum. *JAMA*. 2010;304(8):903-904. doi:10.1001/jama.2010.1208

7. Curtis JR. The Use of Informed Assent in Withholding Cardiopulmonary Resuscitation in the ICU. *AMA J Ethics*. 2012;14(7):545-550. doi:10.1001/virtualmentor.2012.14.7.ecas3-1207

8. Wicclair MR, White DB. Surgeons, Intensivists, and Discretion to Refuse Requested Treatments. *Hastings Cent Rep*. 2014;44(5):33-42. doi:10.1002/hast.356

9. Bosslet GT, Pope TM, Rubenfeld GD, et al. An Official ATS/AACN/ACCP/ESICM/SCCM Policy Statement: Responding to Requests for Potentially Inappropriate Treatments in Intensive Care Units. *Am J Respir Crit Care Med*. 2015;191(11):1318-1330. doi:10.1164/rccm.201505-0924ST

10. Magnus D, Rizk N. Professional Judgment and Justice: Equal Respect for the Professional Judgment of Critical-Care Physicians. *Am J Bioeth*. 2016;16(1):1-2. doi:10.1080/15265161.2016.1128748

11. Wong SPY, McFarland LV, Liu CF, Laundry RJ, Hebert PL, O'Hare AM. Care Practices for Patients With Advanced Kidney Disease Who Forgo Maintenance Dialysis. *JAMA Intern Med*. 2019;179(3):305-313. doi:10.1001/jamainternmed.2018.6197

12. Whitney SN, McCullough LB. Physicians' Silent Decisions: Because Patient Autonomy Does Not Always Come First. *Am J Bioeth*. 2007;7(7):33-38. doi:10.1080/15265160701399735

13. Curtis JR, Burt RA. Point: the ethics of unilateral "do not resuscitate" orders: the role of "informed assent." *Chest*. 2007;132(3):748-751; discussion 755-756. doi:10.1378/chest.07-0745

14. Courtwright AM, Brackett S, Cadge W, Krakauer EL, Robinson EM. Experience with a hospital policy on not offering cardiopulmonary resuscitation when believed more harmful than beneficial. *J Crit Care*. 2015;30(1):173-177. doi:10.1016/j.jcrc.2014.10.003

15. Robinson EM, Cadge W, Zollfrank AA, Cremens MC, Courtwright AM. After the DNR: Surrogates Who Persist in Requesting Cardiopulmonary Resuscitation. *Hastings Cent Rep*. 2017;47(1):10-19. doi:10.1002/hast.664

© 2026 Batten JN et al. JAMA Network Open.

16. Putman MS, D'Alessandro A, Curlin FA, Yoon JD. Unilateral Do Not Resuscitate Orders: Physician Attitudes and Practices. *CHEST*. 2017;152(1):224-225. doi:10.1016/j.chest.2017.03.041

17. Kon AA, Shepard EK, Sederstrom NO, et al. Defining Futile and Potentially Inappropriate Interventions: A Policy Statement From the Society of Critical Care Medicine Ethics Committee. *Crit Care Med*. 2016;44(9):1769-1774. doi:10.1097/CCM.0000000000001965

18. Whitney SN. A New Model of Medical Decisions: Exploring the Limits of Shared Decision Making. *Med Decis Making*. 2003;23(4):275-280. doi:10.1177/0272989X03256006

19. Whitney SN, McGuire AL, McCullough LB. A Typology of Shared Decision Making, Informed Consent, and Simple Consent. *Ann Intern Med*. 2004;140(1):54-59. doi:10.7326/0003-4819-140-1-200401060-00012

20. Whitney SN, Holmes-Rovner M, Brody H, et al. Beyond Shared Decision Making: An Expanded Typology of Medical Decisions. *Med Decis Making*. 2008;28(5):699-705. doi:10.1177/0272989X08318465

21. Drolet BC, White CL. Selective Paternalism. *AMA J Ethics*. 2012;14(7):582-588. doi:10.1001/virtualmentor.2012.14.7.oped2-1207

© 2026 Batten JN et al. JAMA Network Open.
