# Supplementary material for: Decision-Making Approaches Used to Limit Potentially Nonbeneficial Life-Prolonging Interventions
Source: JAMA Netw Open. 2026 Feb 20;9(2):e2560260. doi: 10.1001/jamanetworkopen.2025.60260 (PMC12924098; doi:10.1001/jamanetworkopen.2025.60260)
Supplement: Supplement 2. — Data Sharing Statement [file jamanetwopen-e2560260-s002.pdf]

Data Sharing Statement

Batten. Decision-Making Approaches Used to Limit Potentially Nonbeneficial Life-Prolonging Interventions. *JAMA Netw Open*. Published February 20, 2026.
doi:10.1001/jamanetworkopen.2025.60260

Data
Data available: No

Additional Information
Explanation for why data not available: Given the nature of qualitative research, it is impossible to fully anonymize entire interview transcripts. Thus, we are not able to share interview data while protecting the privacy of research participants. If there are other researchers interested in projects using this data set, they should contact the senior author, Dr. Elizabeth Dzeng, to explore potential collaborations. Any additional collaborators would require IRB approval in order to access the dataset.
